# Spatial Behavior and Habitat Use of Two Sympatric Bat Species

**DOI:** 10.3390/ani11123460

**Published:** 2021-12-05

**Authors:** Nicole Starik, Thomas Göttert, Ulrich Zeller

**Affiliations:** 1 Albrecht Daniel Thaer-Institute of Agricultural and Horticultural Sciences, Faculty of Life Sciences, Humboldt-Universität zu Berlin, 10115 Berlin, Germany; ulrich.zeller@hu-berlin.de; 2 Research Center [Sustainability–Transformation–Transfer], Eberswalde University for Sustainable Development, Schicklerstr. 5, 16225 Eberswalde, Germany; Thomas.Goettert@hnee.de

**Keywords:** *Plecotus*, resource partitioning, radio-tracking, activity areas, fecal analysis

## Abstract

**Simple Summary:**

Few studies refer to ecological differences of genetically close and morphologically almost identical insectivorous bat species. However, this information is indispensable for effective and sustainable nature conservation strategies. This study aims at investigating differences in the spatial ecology of the long-eared bat species *Plecotus auritus* and *Plecotus austriacus* in a typical cultural landscape of Brandenburg, where the two species occur sympatrically. The reconstruction of the prey spectrum revealed that *P. auritus* and *P. austriacus* strongly overlapped in their diet. Our results suggest that resource partitioning is based on using different foraging habitats. While radio-tracked females of *P. auritus* were strongly associated with woodland patches resulting in small-scale activity areas of only few square kilometers, activity areas of *P. austriacus* encompassed a large-scale matrix of grassland habitats in the magnitude of a small town. Based on these results, we identify priority conservation needs for the two species to ensure that these differences in the spatial behavior and habitat use can be adequately taken into account for future nature conservation efforts.

**Abstract:**

Movement behavior and habitat use of the long-eared bat species *Plecotus auritus* and *Plecotus austriacus* were studied in the Havelland region in Brandenburg (Germany). Data collection included mist-netting, radiotelemetry, reconstruction of prey items, and monitoring of roosting sites. Body measurements confirm a high degree of phenotypic similarity between the two species. Total activity areas (100% Minimum Convex Polygons, MCPS) of *Plecotus austriacus* (2828.3 ± 1269.43 ha) were up to five-fold larger compared to *Plecotus auritus* (544.54 ± 295.89 ha). The activity areas of *Plecotus austriacus* contained up to 11 distinct core areas, and their mean total size (149.7 ± 0.07 ha) was approximately three-fold larger compared to core areas of *Plecotus auritus* (49.2 ± 25.6 ha). The mean distance between consecutive fixes per night was 12.72 ± 3.7 km for *Plecotus austriacus* and 4.23 ± 2.8 km for *Plecotus auritus.* While *Plecotus austriacus* was located most frequently over pastures (>40%) and meadows (>20%), *P. auritus* was located mostly within deciduous (>50%) and mixed forests (>30%) in close vicinity to its roosts. Roost site monitoring indicates that the activity of *P. austriacus* is delayed relative to *P. auritus* in spring and declined earlier in autumn. These phenological differences are probably related to the species’ respective diets. Levins’ measure of trophic niche breadth suggests that the prey spectrum for *P. auritus* is more diverse during spring (B = 2.86) and autumn (B = 2.82) compared to *P. austriacus* (spring: B = 1.7; autumn: B = 2.1). Our results give reason to consider these interspecific ecological variations and species-specific requirements of *P. auritus* and *P. austriacus* to develop adapted and improved conservation measures.

## 1. Introduction

Species that share habitats as well as ecomorphological features due to adaptive convergence or phylogenetic proximity pose a challenge to conservationists and require enhanced conservation strategies [1,2]. Accordingly, considerable attention has been given to understanding variations in the ecology among closely-related co-occurring (sympatric) species [3,4,5], which is an essential precondition to develop appropriate conservation policies [6,7].

Usually, the coexistence of sympatric species is facilitated by sufficient competition-driven ecological differentiation [8,9], in most cases through the division of limiting resources (resource partitioning). According to the competitive exclusion principle, this partitioning of the ecological niche is the fundamental mechanism that allows co-occurring species to coexist in the same environment [10,11]. Where the niches of two species are highly similar (i.e., high degree of niche overlap), out-competing can occur if the resource in question is limited. Thus, morphologically similar (and ecologically alike) species can evolve intense interspecific competition due to a comparably low degree of niche differentiation [12]. In most cases, competing species overlap in various aspects of their biology with only minor, but still qualitative, differences in their ecological niches (e.g., roosting behavior, habitat preferences). This can complicate current approaches to define reasonable conservation measures. Usually, animals are not limited by only a single resource, but rather by a multitude of abiotic and biotic factors [13]. However, there are examples of co-occurring species that are indeed primarily limited by only a single resource [14], and recent studies indicate that nature conservation measures for such species do not take this into account sufficiently. 

Long-eared bats of the genus *Plecotus* are widely distributed and common over most of the Palaearctic [15]. Recently, the genus has undergone remarkable taxonomic revision [15,16,17,18]. Based on molecular evidence, a complex of several cryptic species within two major clades exists with seven European species being described to date [19,20,21,22,23,24,25]. However, phylogenetic relationships and phylogeographic patterns of the genus are still not fully understood due to non-comprehensive sampling. Recent phylogenetic evaluations of morphological and karyological data support that the brown long-eared bat *Plecotus auritus* (Linnaeus, 1758) and the grey long-eared bat *Plecotus austriacus* (J. Fischer, 1829) widely co-occur in central Europe. In northern Germany, *P. austriacus* is at the northern edge of its distribution range and can be regarded as one of the rarest bat species in the region. The two species are morphologically very similar assuming similar ecological behavior and niche breadth. For the assignment of individuals to either species, an existing set of morphological and skull characters can be used [21,26,27]. Several authors have investigated aspects of ecology, and habitat requirements of the two species seem well documented. The typical forest-associated species *P. auritus* is mainly a foliage gleaner [27]. *P. austriacus*, adapted to more open habitats, is known to glean insects from vertical surfaces, such as buildings and rocks, and from leaves, but is also a slow aerial hawker [27].

However, most of the available literature is focused on either *P. auritus* or *P. austriacus* and only few studies investigated the ecology of these closely-related species when occurring in syntopy (occupying the same locality at the same time). We think that these contact zones of sympatric occurrence are especially interesting as they can enhance our understanding of the coexistence of similar species within the same habitat. *P. auritus* and *P. austriacus* offer a suitable example for investigating variations in the ecology of two sympatric bat species representing the same guild. Given that there is resource partitioning between these closely-related species, more species-specific conservation and monitoring strategies may become necessary. In addition, such information can greatly contribute to the current knowledge on the response patterns and sensitivity of these species towards specific land management practices and helps to evaluate their suitability as ecological indicators based on their organismic capabilities and limitations [28,29,30].

Our overall objective is to investigate possible ecological differences between the two long-eared bat species *P. auritus* and *P. austriacus* in a typical cultural landscape in Germany, where both species are known to occur in sympatry. The first objective is to examine the distribution of the two species in the study area and to compare external morphological features suggested in the literature to ascertain species identification. The next objective is to analyze the spatial behavior (activity areas, core areas, travel distances) and habitat use of selected individuals representing different colonies. In a final step, we aim at analyzing the relations between spatial behavior, habitat use, diet and phenology of the two species. Collectively, a better understanding of these relations will help to derive future management implications for the conservation of long-eared bats and a sustainable land management, which addresses the requirements and ecological function of sympatric bat species in this particular cultural landscape.

## 2. Materials and Methods

### 2.1. Study Area

**Figure 1:**
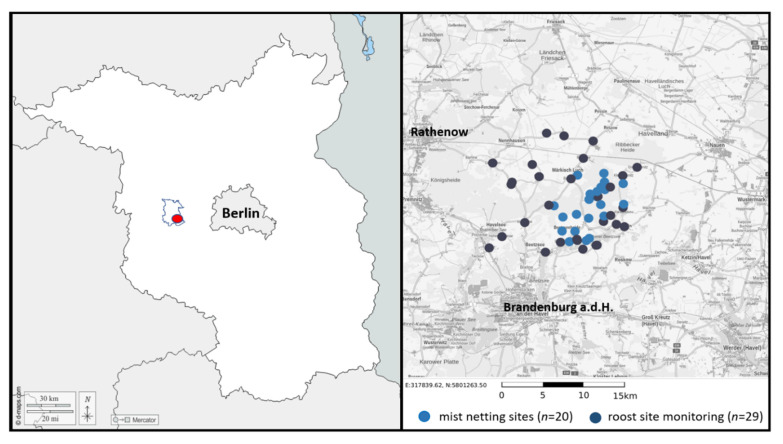
**Left**: Location of the study area (red dot) covering an area of approx. 100 km^2^ within the Havelland (blue line). **Right**: Presence and distribution of *P. auritus* and *P. austriacus* in the study area was surveyed by systematically capturing bats with mist-nets at selected study sites (*n* = 20) and systematically searching in possible roosting sites (i.e., churches, large agricultural holdings, and private houses; *n* = 29) between May and October 2012 and 2013.

The study was conducted in the nature park Westhavelland (52°40′41″ N, 12°15′3″ O), which is located approximately 70 km west of Berlin (Figure 1). The nature park Westhavelland is a typical cultural landscape which has been subject to human influences at least since 9500 B.C. It is dominated by meadows, riparian lowlands, forests and arable land, interspersed with small villages and riverine landscapes. With approximately 22–63 m asl, the area is defined by its waters, especially those of the River Havel with its distinctive lowlands (Lower Havel river basin). The average annual rainfall is 550 mm. In the lowlands, the strongly fluctuating water levels over the course of the year, the presence of different soil types, and especially the shallow relief of the soil, create a variety of different site conditions. In combination with the different forms of land use, this led to characteristic forms of vegetation, among them are many species on the Red List, e.g., Siberian iris, marsh gentian, rattle-brain, and dyer’s orchid. Large areas of the nature park are landscape conservation areas (LSG). Particularly representative areas are designated as nature conservation areas (NSG) and are anchored in the network of European protected areas Natura2000 under the Flora–Fauna–Habitat Directive (FFH) and bird sanctuaries (SPA). Thus, the nature park is of great importance as a protected area for many Nordic migratory birds.

### 2.2. Data Collection

In order to detect the two species in the study area and to investigate details of their ecology, we used several methods including mist-netting, acoustic monitoring, monitoring of roosting sites, analysis of fecal pellets and feeding remains, and radio-tracking between 2013 and 2015. 

#### 2.2.1. Species Occurrence and Phenotypic Distinction

We confirmed the occurrence of the two species by systematically trapping at twenty different study sites (woodland/open space) covering an area of approx. 100 km^2^ that represented all major habitat types and land use forms: intensively managed agricultural land, semi-improved and unimproved acidic and calcareous grassland, woodland, and a variety of human settlements. Bats were captured during full nights from sunset to dawn using ground-level (2.9 m) and canopy (10 m) mist-nets combining units of different lengths (3−23 m) during a total of 196 nights. Total sampling effort was 117,626 m^2^ net-hours.

Bats were identified in the field using reliable biometric and morphological characteristics [31] and recognized as adults or juveniles by inspecting epiphyseal fusions of the bats’ forearms. Only adult bats were measured. The following measures were taken with a mechanical precision caliper: head body length (HBL), thumb length (TL), hindfoot length (HFL), claw length (CL), ear length (EL), tragus length (TrL), tragus width (TrW), forearm length including wrist (FA), length of the third finger excluding wrist (D3), and length of the fifth finger excluding wrist (D5). Handling of captured animals was done in accordance with the Guidelines from the American Society of Mammalogists.

As morphological traits are crucial for flight performance and foraging ecology in bats, we investigated possible minute differences in wing morphology of captured individuals of both species. Using the criteria laid out by Norberg and Rayner [32], wingspan (B), wing area (S), wing loading (WL), and aspect ratio (AR) of the bats were calculated. The interrelationships of these parameters allow predictions to be made about the preferred habitat of a species. The wing-span (B) is the distance between the two wing tips. This was obtained by the measured distance of the left wing (=wing length), starting proximal to distal, to the tip. However, the actual wing-span is about 10% larger than the simply combined length of two wings [32]. This length (10%) was therefore added to the determined measurement distances. According to Blood and McFarlane [33], the wing area was further calculated as:

S = (FA × D5) + 0.5 × (D5 × D3)(1)

where S = wing area [m^2^], FA = forearm length [mm], D5 = length of fifth finger [mm], and D3 = length of third finger [mm].

However, since the wing area still includes parts of the body and possibly the tail [32], 16% were added to the calculated wing area according to Dwyer [34], Jones and Suttkus [35] and Nicoll and Suttie [36]. The wing aspect ratio (AR) could now be calculated as follows:

AR = B × B/S(2)

where AR = aspect ratio, B = wing-span, and S = wing area.

Finally, wing loading (WL) was calculated according to Norberg and Rayner [32] as follows:

WL = (m × g)/S(3)

where WL = wing loading, m = body weight, g = 9.81 ms^−1^, and S = wing area.

To assess morphological variation between species, we calculated the mean, SD, minimum, and maximum for morphological variables. One-way analysis of variance (F-ANOVA) with post-hoc tests for homogeneous groups (Tukey’s HSD) were performed for those variables with normal distribution. For variables without normal distribution, non-parametric Kruskal–Wallis (H-KW) was used, and multiple comparisons were performed by the method of Dunn. In both, Bonferroni corrections for multiple comparisons were included [37]. To visualize how individuals belonging to each species are grouped according to their morphological affinities and to identify the characters that best define the groups, principal component analysis (PCA) was performed with captured individuals and considering all calculated external morphological characters. Contributions of the variables (correlation values) to each principal component (PC) were interpreted as significant when values of factor loading were greater than 0.6. Graphs were constructed with axes corresponding to the most informative PCs. 

#### 2.2.2. Phenology, Activity and Emergence Behavior

To obtain information on the phenology and timing of activity of *P. auritus* and *P. austriacus,* we compiled data collected during mist-netting (data on species occurrence within study region), automatic continuous recording of echolocation calls, and emergence counts at two different maternity colonies. We assumed that the recorded data, regardless of the methods, were correlated with the actual bat activity. Exact time of capture events of the two species during mist-net sampling was used to describe activity patterns. Ultrasonic recordings of bat calls were made at colony entrances one hour prior to sunset until one hour after sunrise (Linde, Germany, 52°32′ N and 12°39′ E) between April and November 2014. Both colonies were identified during roost detection permitting additional and continuous visual species identification of roosting individuals during daytime on several occasions. Using roost sites with only one and not several entrances, we made sure to reliably detect roost leaving or entering individuals. 

Batcorder (ecoObs GmbH, Nuremberg, Germany) were installed close to the roost entrances of the colonies (5 m maximum distance) to record ultrasonic calls of bats. BatSound (Petersson Electronics, Uppsala, Sweden) was used to measure call parameters and to validate species identification. The final diagnosis of the bat calls was mainly based on the course frequency, start and end frequency, main frequency, and call length accordingly [38]. We caution that acoustic discrimination of the two species is extremely difficult, however still possible. Using only calls of the same call length, the species differ in the start frequency and in the maximum frequency of the first harmonic by 5−10 kHz [39,40,41]. In principle, this also applies to the second harmonic. These frequencies vary considerably depending on the call length [38], which is why only the latter should be the basis for assessment, as otherwise false species determinations may result. In addition, both species regularly emit species-specific social calls at summer roost sites around the time of entering and exiting the roost [42,43] and during dawn swarming at roost sites. Together with the visual species identification at the roost sites and emergence counts using night vision equipment, we were able to ensure that the acoustic recordings could be attributed to either *P. auritus* or *P. austriacus*. Thus, we are confident that our observations did not include species other than *P. auritus* or *P. austriacus*.

#### 2.2.3. Reconstruction of the Prey Spectrum

We used microscopic analysis of feces collected at identified day roosting sites of *P. auritus* and *P. austriacus*. The analysis of bat feces provides an approximate insight into the food spectrum of bats. Compared to molecular biological analyses, the method may not reflect the exact composition of the ingested food, but it allows a good estimation of the abundance of different prey groups and is thus suitable for both seasonal and geographical comparisons with regard to the food composition of native bat species. In order to describe the diet of the two species in more detail, we aimed at the identification of lepidopteran families from collected feeding remains under selected feeding perches identified by radio-tracking (see below). Feeding perches are generally night roosts used to consume insect prey transported from nearby feeding areas in order to avoid energy-costly commuting to day-roosts, but day-roosts can also be used. 

To identify day-roosts and feeding perches of *P. auritus* and *P. austriacus*, we surveyed the study area to identify any direct evidence of these species such as roosting individuals, bat droppings or feeding remains by systematically searching in churches (*n* = 17), large agricultural holdings (*n* = 4) and private houses (*n* = 8) between May and October 2012 and 2013. Feeding perches have been found in churches and houses and were confirmed by the presence of accumulations of insect remains and bat droppings. Other species that may also feed extensively on Lepidoptera and possibly use similar feeding-perches like those of *P. auritus* or *P. austriacus*, are Natterer’s bat *Myotis nattereri*, Bechstein’s bat *M. bechsteinii*, and possibly the barbastelle *Barbastella barbastellus*, but none of these were detected during roost site inspections. Thus, we are confident that our samples did not include feces and food remains from species other than *P. auritus* or *P. austriacus* respectively. Polyethylene films (3.9 m × 4.9 m) were used to collect fecal samples (2013, 2014) and feeding remains (2013–2015) between April–November under identified hanging places. The film was renewed/emptied every 4 weeks to assure representative data collection across seasons. For *P. auritus*, we collected a total of 43 samples from three different colonies (two churches, one house), and for *P. austriacus,* 24 samples from two different colonies (one church, one house). Fecal samples were stored in sample tubes at −20 °C. For microscopic analysis, a random selection of 10 pellets from each sample was used for further evaluation, resulting in 430 analyzed pellets for *P. auritus* and 240 analyzed pellets for *P. austriacus*. Each pellet was analyzed separately, dissoluted in Petri dishes in water and a drop of glycerol (to achieve better distribution of fragments). The droppings were teased apart and processed with dissecting needles. Subsequent determination of prey fragments was achieved under a stereomicroscope (Olympus SZ2-ILST) with 6–48 magnification according to Beck [44]. 

Arthropod fragments have been identified at least to order level, some (especially Lepidoptera) could be identified to family level using comparative slides, methodological works [45,46,47], entomological keys, and reference material collected in the field (insect catches light traps/barber traps). We determined mean volume proportion (%V), numerical proportion (%N), and frequency of occurrence (%O) for each prey item in the droppings. Percentage volume (%V) was assessed according to Obrtel and Holišová [48] and averaged for each sample (collection date, removal of foil at roosting sites) to provide an index of the proportional contribution of insect taxa to the diet of the species [49]. We defined a prey item as predominant if it comprised more than half of total volume of a sample. The frequency of occurrence, ranging from 0–100%, provides a standardized measure of the commonness and thus, relative importance of each prey item in the diet [46]. Frequency of occurrence is the number of fecal samples in which a particular prey item was identified, divided by the sum of the numbers of samples that contained each identified prey item [46]. We used the arithmetic mean of the frequency of occurrence and percentage of volume to estimate relative importance of each prey item [49]. Significant differences in the order-level identifications based on frequency of occurrence data were tested using a Wilcoxon Mann–Whitney test. Differences were considered significant at the 0.05 level. The trophic niche breadth as well as seasonal changes in dietary diversity were calculated according to the formula proposed by Levins [50]:

(4) $$B=\frac{1}{\sum p_{j^{2}}}=\frac{Y^{2}}{\sum n_{j^{2}}}$$

where *B* = Levins’ measure of niche breadth; *p_j_* = the proportion of individuals found in or using resource state *j*, or the fraction of items in the diet that are of food category *j* (estimated by *n_j_*/*Y*) (Σ*p_j_* = 1.0); *n_j_* = the number of individuals found in or using resource state *j*; *Y* = Σ*n_j_* = the total number of individuals sampled. For the analysis of overlap in niche dimensions and/or the degree of similarity between the diets, we used Pianka’s niche overlap index (*O_jk_*) with values ranging from 0 (no overlap) to 1 (complete overlap) [51]:

(5) $$O_{jk}=\sum_{n=1}^{o}p_{ij}\times p_{ik}\;/\sqrt{\sum_{n=1}^{o}p_{ij}{}^{2}}\times \sum_{n=1}^{o}p_{ik}{}^{2}$$

where *O_jk_* represents the index of overlap of Pianka’s niche between species *j* and *k*; *p_ij_* is the proportion of resource *i* in total resources used by species *j*; *p_ik_* is the proportion of resource *i* in total resources used by species *k*; and *n* is the total number of resource categories for species *j* and *k*. 

We analyzed feeding remains from a total of four identified feeding perches of *P. auritus* (*n* = 27 sampling dates) and three feeding perches of *P. austriacus* (*n* = 24 sampling dates) and calculated the numerical proportion of lepidopteran families in each sample. Nearly all remains found during our study were represented by wings of nocturnal moths; in several instances we found parts of the prothorax of noctuids with conjoined anterior wings and legs.

#### 2.2.4. Habitat Use and Foraging Behavior

We used radio telemetry to address the spatial distribution, activity area sizes and foraging characteristics of the two species. Out of *n* = 94 bats captured on several occasions between May 2013 and October 2015 (see above), each five female lactating bats of *P. auritus* and *P. austriacus* were radio-tracked during maternity seasons. A small patch of hair was removed from the back of the bats, between the scapula, to allow the transmitters to be fixed in place with the adhesive. Bats weighing 8–12 g were fitted with 0.35 g radio transmitters (PipII, Biotrack Ltd., Dorset, UK) and released at the site of capture. The transmitters weighed on average 6% of the mass of both species (range 4.9–7%). An ATS scanning receiver (Model R2100, Advanced Telemetry Systems, Isanti, MN, USA) connected to a three element, hand-held, Yagi antenna was used to track the bats. Bats were tracked and located by using continuous tracking methods [51,52] as often as accessibility made it feasible, for an average of 6.4 nights for *P. auritus* and 5.0 nights for *P. austriacus*. In the first hours after tagging, we only followed the bats to identify their day-roosts in order to start tracking from emergence from the roost the following night and we did not include locations from the first night into the subsequent activity area analysis. Based on the species’ reported foraging range, a radius of approx. 10 km from these roosting locations was surveyed during the following nights to search for signals from bats in roosts, mainly on foot in areas far from roads or vehicle tracks. Each bat’s location (±15 m) was recorded every 15 min to allow for the calculation of activity areas, core areas and habitat use. Fixes were acquired using a “homing in” method [53]: the observer approached a tagged bat as closely as possible and when the signal was very strong, a compass bearing and distance (based on signal strength) were recorded. However, if the bat was stationary (roosting), its exact location (±3 m) was recorded using a hand-held GPS unit (Garmin Etrex, Garmin International Ltd., Olathe, KS, USA). 

Individual activity area sizes (ha) were estimated per night with Ranges VI (Anatrack; Wareham, Dorset, UK). To define core (foraging) areas, we analyzed utilization distribution discontinuities [54]. This analysis indicates the point where outlying fixes are excluded [55,56,57] by plotting the polygon against the percentage inclusion of locations, allowing the creation of cluster cores that represent foraging areas. Although several methods are available to define core foraging areas of animals [58,59,60], cluster polygons (cores) were considered the most appropriate minimum-linkage estimators to define the core areas in which bats foraged. Furthermore, locations collected from each individual could not be assumed independent enough for location density estimators of activity areas that make parametric assumptions [61,62]. For both species, cores had been estimated for 98% of the ranges when 15% of the locations were removed, meaning that the excursion excluded core area contained 85% of the locations. Examination of removed fixes revealed that they were primarily recorded as bats commuted from roosts to foraging areas. Thus 85% cluster cores were used to assess the habitat in which bats were foraging. We used Quantum GIS and CIR biotope type data (approx. 2500 biotope type classes) for map representation. Commuting, foraging and resting behavior were classified according to Entwistle et al. [63]. For each individual bat and night, the distances travelled were measured. We created radii around the identified roosting sites to assess frequency distribution of location within a given buffer radius and to estimate average distance recorded between roosting sites and foraging habitats. To investigate whether there was a difference in average activity area size, core area, travel distance per night and maximum distance to foraging areas, we log-transformed data to obtain normality and used an independent two sample t-test.

## 3. Results

### 3.1. Comparison of External Characteristics

**Figure 2:**
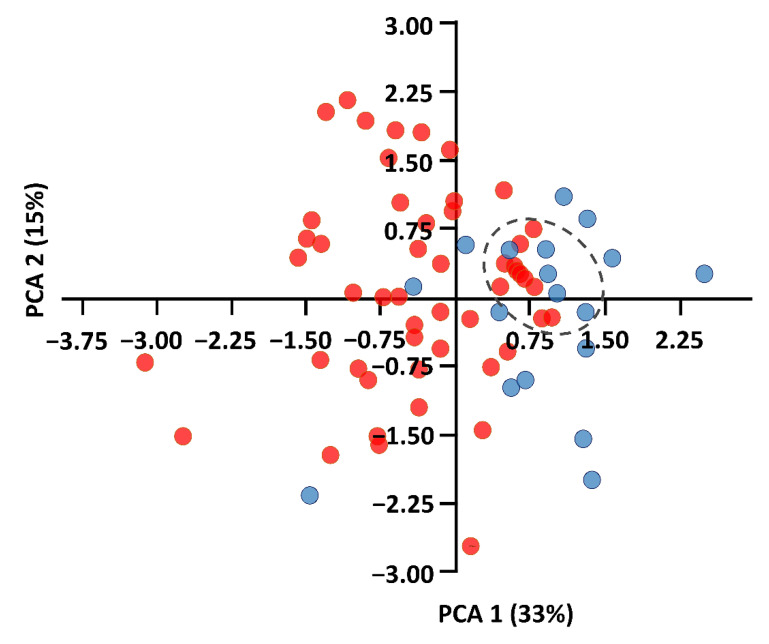
PCA scatterplot of the first two axes (PC1 and PC2) of 51 individuals of *P*. *auritus* (red) and 26 individuals of *P*. *austriacus* (blue) based on 15 measured external characters. Percentage of total variance associated to each PC is provided in parentheses. Among the measured individuals of *P. auritus* (red), 10 males show a high degree of similarity among each other and in relation to some individuals of *P. austriacus* (dotted circle).

Body measurements were obtained from 51 adults of *P. auritus* (16 male, 35 female) and 26 adults of *P. austriacus* (12 male, 14 female). ANOVA and Kruskal–Wallis analyses reveal significant differences for 10 out of 15 measurements (Table S1). This was especially pronounced for the third (*p* = 0.02) and fifth (*p* = 0.04) finger. The PCA scatterplot shows a substantial overlap between *P. auritus* and *P. austriacus* along the two axes. The first two components account for 48% of the total variation of the data, with PC1 representing 33%, PC2, 15%, and PC3, 9% (Figure 2). Individuals of *P. austriacus* form a homogenous group of data within the plot (blue). Among the measured individuals of *P. auritus* (red), 10 males show a high degree of similarity among each other and in relation to some individuals of *P. austriacus* (marked with dotted circle). The characters that contributed most to PC1 are mainly related to wing measurements and flight performance (characters FA, D3, D5, WS, S, AR, WL; Table S1), and the characters that contributed most to PC2 are mainly related to TL, CL, TrL, Trw (Table S1). 

### 3.2. Spatial Behavior and Habitat Use

**Figure 3:**
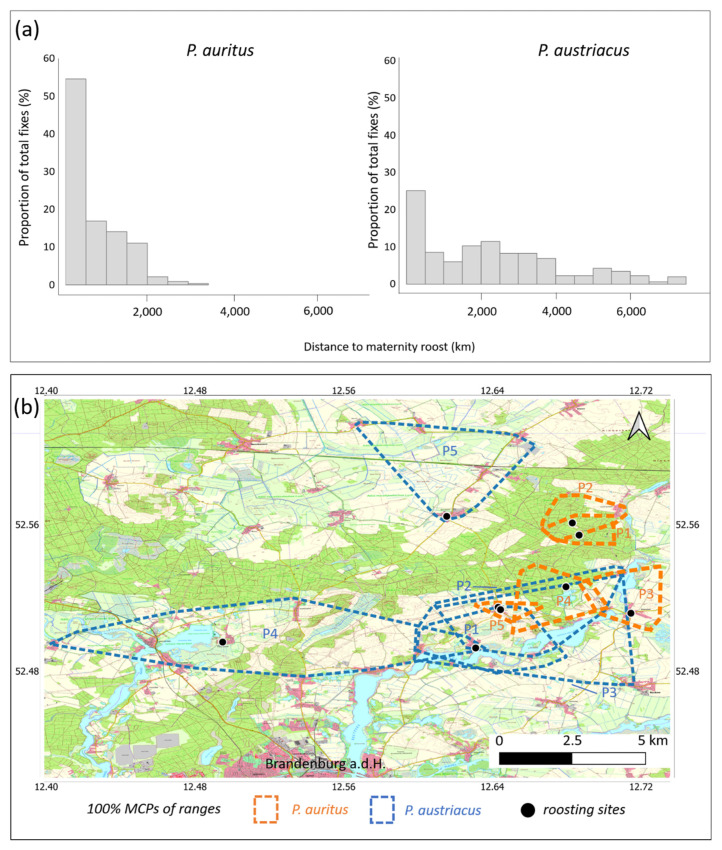
(**a**) Frequency distribution of recorded distances from the maternity colony of each five female *P. auritus* (*n* = 332 locations, left) and *P. austriacus* (*n* = 362 locations, right); (**b**) range outlines (100% MCPs) of *P. auritus* (*n* = 5 females, orange) and *P. austriacus* (*n* = 5 females, blue) radio-tracked in the nature park Westhavelland during 2013−2015.

**Figure 4:**
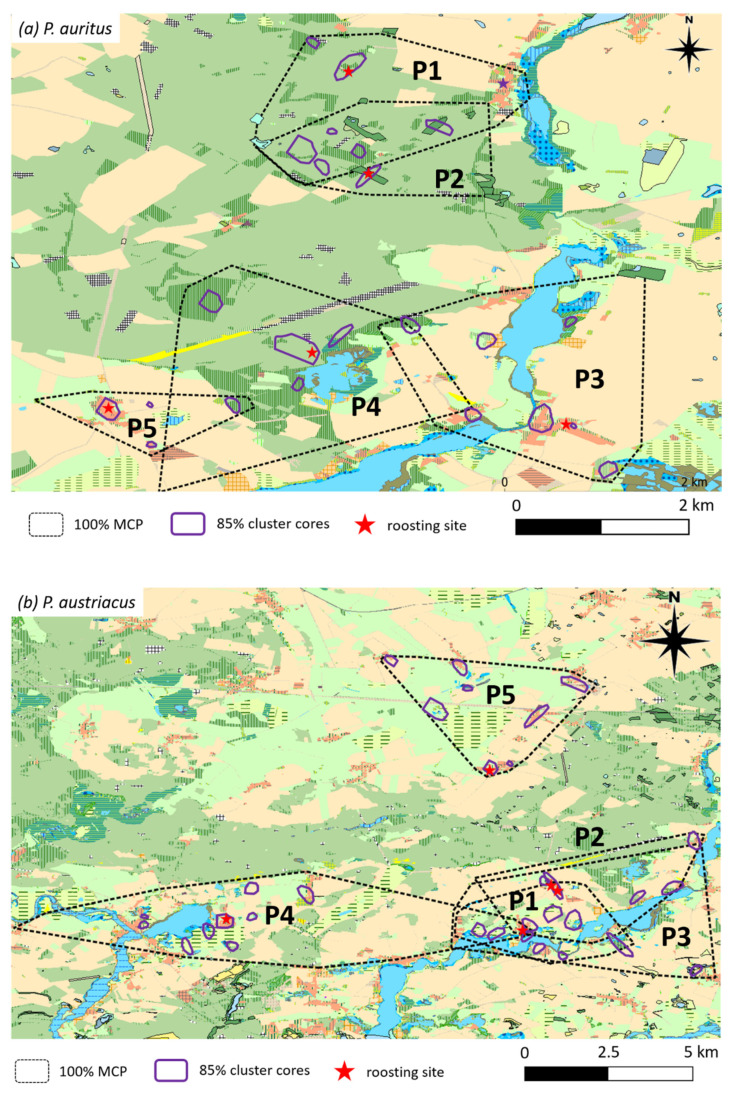
MCPs and 85% cluster core areas of female *P. auritus* (**a**) and *P. austriacus* (**b**) radio-tracked during maternity seasons between 2013 and 2015 (**a** and **b** respectively; *n* = 5 females).

Data was obtained from each five female individuals per species during 2013−2015 (Table 1). A total of *n* = 332 (*P. auritus*) and *n* = 362 (*P. austriacus*) fixes had been acquired, with a mean of 10.4 ± 3.89 (*P. auritus*) and 13.8 ± 5.41 (*P. austriacus*) fixes per individual per day. Over half of the locations of radio-tracked *P. auritus* were within a radius of 500 m from the roost (55%) and the maximum distance to the roost recorded was 3.5 km (Figure 3a). In contrast, only 25% of the locations of *P. austriacus* were within a radius of 500 m from the roost and the maximum distance to the roost recorded was 7.5 km (Figure 3a). With a five-fold of the size, *P. austriacus* showed significantly larger activity areas (100% MCP) compared to *P. auritus* (*p* < 0.001; Table 1, Figure 3b.). For both species, core foraging areas (85% cluster cores) were visibly smaller than the total MCP through which individual bats travelled (Figure 4). Core areas were approximately three-fold larger for *P. austriacus* compared to *P. auritus*. In addition, mean travel distance per night (*p* < 0.001) and mean distance from roost to foraging areas were significantly larger for *P. austriacus (p* < 0.001).

**Figure 5:**
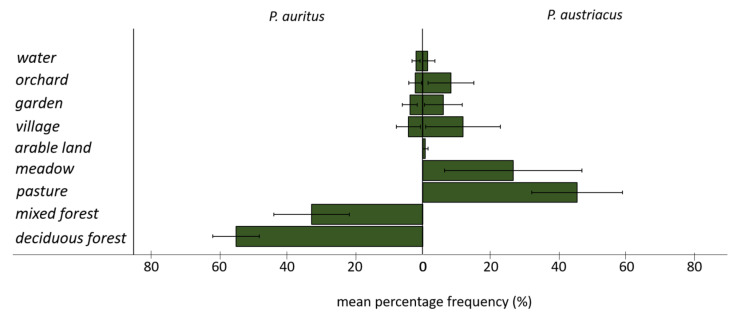
Mean percentage frequency of utilization of the main habitat categories by radio-tracked female *P. auritus* (*n* = 332 locations, left) and *P. austriacus* (*n* = 362 locations, right).

Whereas the 100% MCPs encompass up to twenty different habitat types for both species, core areas include only five (*P. auritus*) and seven (*P. austriacus*) different habitat types (Figure 4). The two species differed in the mean percentage frequency of locations in different habitat types (Figure 5). While *P. auritus* was observed almost exclusively in deciduous (>50%) and mixed forests (>30%), the majority of locations of *P. austriacus* was in the grassland dominated matrix, most frequently over pastures (>40%) and meadows (>20%) (Figure 4 and Figure 5). None of the radio-tracked individuals of *P. auritus* have been located in open agricultural areas at any time. However, some habitat types were also used by both species; e.g., villages with 5% (*P. auritus*) and 15% (*P. austriacus*), orchards with 3% and 8%, private gardens with 4% and 6%, and water bodies with 2% and 1% of the respective species’ total locations.

### 3.3. Reconstruction of the Prey Spectrum

**Figure 6:**
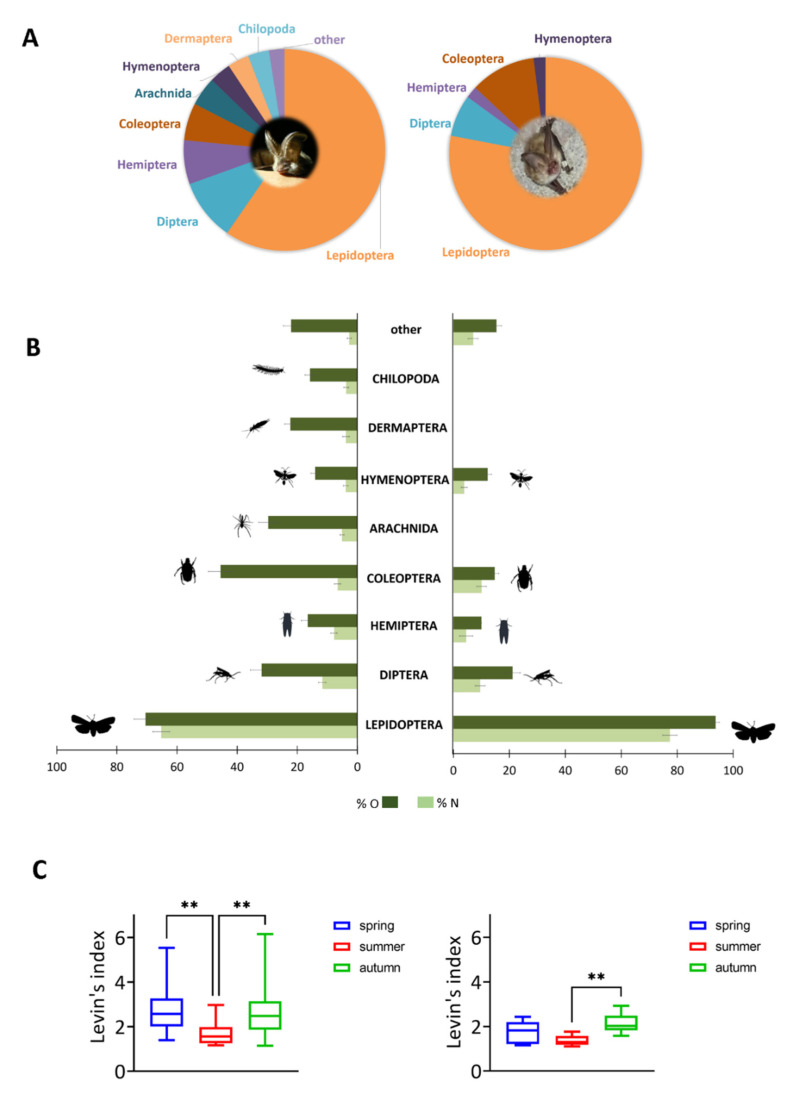
Reconstruction of prey spectra of *P. auritus* (left) and *P. austriacus* (right). (**A**) Mean volume proportion of prey items at the ordinal level. (**B**) Mean frequency of occurrence (%O) and numerical abundance (%N) of prey items (mean ± SD) at the ordinal level determined by microscopic fecal analysis. (**C**) Seasonal changes of Levins’ trophic niche breadth index B. Significant differences between seasons are indicated by asterisks (** *p* ≤ 0.005). *P. auritus: n =* 430 pellets, *P. austriacus: n =* 240 pellets.

Microscopic fecal analysis indicates a high overlap between the two species regarding the proven prey categories at the ordinal level considering volume (%V) and numerical (%N) proportion (Figure 6). Pianka’s overlap index between the species is 0.81. With regard to volume proportion, Lepidoptera constitute 65% (*P. auritus*) and 77% (*P. austriacus*) of the fecal samples investigated (Figure 6A). However, with regard to frequency of occurrence (%O) and numerical abundance (%N) of prey identifications, there is a significant difference for the consumption of Lepidoptera in the diet of *P. auritus* and *P. austriacus* (*p* < 0.005) (Figure 6B). Dipterans and beetles could be found in samples of both species. While in samples of *P. auritus,* arachnids, earwigs (Dermaptera), and myriapod arthropods (Chilopoda) were frequently detected, these prey items could not be detected in the fecal samples of *P. austriacus*. In general, *P. auritus* is characterised by a significantly larger dietary niche breadth compared to *P. austriacus* (*p* < 0.005). Niche breadth for *P. auritus* varies from 1.13 in autumn to 5.5 in spring. In contrast, seasonal niche breadth varies from 1.1 (summer) to 2.9 (autumn) for *P. austriacus*. Significant interseasonal differences were found for *P*. *auritus* between spring and summer, as well as between summer and autumn. For *P. austriacus,* seasonal niche breadth considerably differs between summer and autumn (Figure 6C).

**Figure 7:**
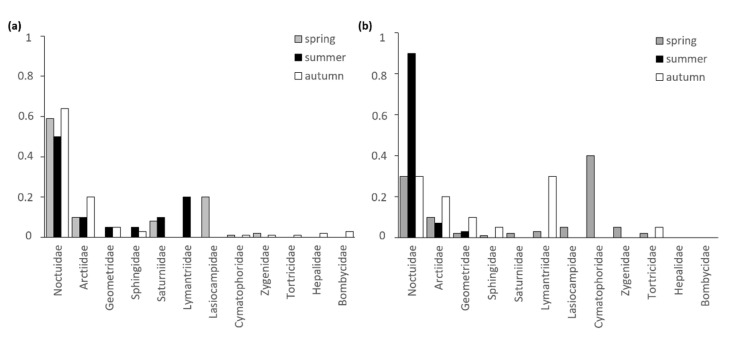
Seasonal proportion of lepidopteran families in samples of feeding remains collected under feeding perches of (**a**) *P. auritus* (*n* = 27 sampling dates) and (**b**) *P. austriacus* (*n* = 24 sampling dates).

For both species, the lepidopteran families Noctuidae and Arctiidae could be detected most frequently in the samples of feeding remains (Figure 7). While Noctuidae play a role in the diet of *P. auritus* during all seasons, this lepidopteran family constitutes a nominal part for *P. austriacus* only during the summer months. In addition, Saturniidae and Lymantriidae make up a notable proportion in the feeding remains of *P. auritus* during the reproductive season. In contrast, moths of the family Cymatophoridae make up a notable proportion in the feeding remains of *P. austriacus* during spring. Nocturnal moths from the family Arctiidae were found in samples of both species with the highest proportion in autumn. 

### 3.4. Seasonal and Nocturnal Activity Patterns

**Figure 8:**
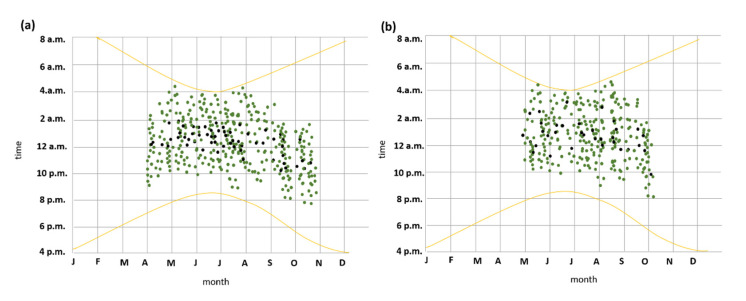
Annual and nocturnal distribution of activity of *P. auritus* (**a**) and *P. austriacus* (**b**). Green dots show time of recordings at two distinct maternity colonies in 2014; black dots show time of all mist-netting capture events of individuals between 2012–2014. The yellow curve lines mark sunset and sunrise (Central European Time, CET or Coordinated Universal Time, UTC + 1).

Both recordings and captures indicate that seasonal phenology of species activity of *P. austriacus* was delayed relative to *P. auritus* in spring and ended earlier in autumn (Figure 8). *P. auritus* were observed from April to the end of October, whereas recordings and captures from *P. austriacus* were recognized from May to the beginning of October only (Figure 8). However, both species exhibit similar nightly emergence patterns. Throughout maternity season (May–August), the first individuals leaving the roost of both species were detected approximately 30 ± 10 min (*P. auritus*) and 25 ± 10 min (*P. austriacus*) after sunset and the last individuals returning to the roost were detected approximately half an hour before sunrise at the latest. However, foraging time spent per night was significantly longer for *P. austriacus* compared to *P. auritus* (*p* < 0.05), while night roosting was significantly shorter (*p* < 0.05).

## 4. Discussion

Despite their high degree of ecomorphological similarity, studied females of the two long-eared bat species *P. auritus* and *P. austriacus* show remarkable differences in their spatial behavior and habitat use during reproductive season: total activity areas of *Plecotus austriacus* were up to five-fold larger compared to *Plecotus auritus*. The activity areas of *Plecotus austriacus* contained up to 11 distinct core areas which were approximately three-fold larger compared to core areas of *Plecotus auritus*. *P. austriacus* foraged up to 6 km from its roosts, which could be found exclusively in buildings. While *P. austriacus* used mostly pastures and hedges in a grassland-dominated matrix, *P. auritus* was located mostly within patchy deciduous and mixed forests in close vicinity (≤500 m) to its roosts. Our activity area estimations for brown long-eared bats correspond with literature data [27]. However, the activity areas measured for grey long-eared bats are much larger than the areas reported from other studies, e.g., 75 ha [63] or 12.9−803.96 ha [64]. Given the minor differences in body measurements and the high degree of phenotypic similarity of the two species (see Figure 2, Table S1), this tremendous behavioral difference appears very interesting and raises a number of questions. For example, how do the species realize such different spatial strategies and feeding habits given their similar organismic features, and what could be the underlying mechanism?

One explanation could be the competition exclusion principle, according to which similar species evolve different ecological niches to reduce or avoid competition [10,11]. Partitioning of habitat and of diet are important factors in bat niche separation [6,7]. Foraging ecology plays a major role in this context, because food acquisition is essential for survival. When sympatric species show similar foraging mechanisms, they face the problem to minimize niche overlap, especially when prey is limited. Separation can be accomplished by several mechanisms, including morphological variation [65,66,67,68], differences in sensory ecology [69], selection of different habitats, prey types, foraging times, and foraging styles, e.g., [69,70,71].

We think that in our study, the most likely mechanism of niche segregation of sympatric *P. auritus* and *P. austriacus* is the partitioning by habitat use; despite occupying similar roosting places in houses or churches, *P. auritus* and *P. austriacus* showed quite different patterns of habitat use during our study. Their primary foraging habitats are species-specific, with *P. auritus* using mainly mixed and deciduous forests, whereas foraging habitats of *P. austriacus* were associated with trees adjoining improved grassland (pasture land and meadows). While in other studies, both species have been reported to forage in woodland [63,72,73], *P. austriacus* was not located within woodland during our study. In order to make use of the grassland habitat available in the study area, *P. austriacus* needs to cover longer distances to reach suitable foraging sites compared to *P. auritus*. This is also confirmed by the different nocturnal activity patterns observed in our study. Although the observed times of emergence are similar for both species (30 ± 10 min after sunset for *P. auritus* and 25 ± 10 min for *P. austriacus*), foraging time spent per night was significantly longer for *P. austriacus* (347 ± 117 min) compared to *P. auritus* (311 ± 135 min). This finding may relate to the movement activity of the species. Distances of seasonal inter-colony movements of *Plecotus auritus* measure rarely more than 1 km [72,73,74]. In contrast, *Plecotus austriacus* is more mobile, flying fast and straight in open habitats [75], with its longest movements between summer and winter colony roosting sites of approximately 18 km [64] to 62 km [76]. Moreover, this finding could relate to different dietary patterns influencing the spatial range needed to reach suitable foraging sites. 

In a previous study, Ashrafi et al. [6] showed that *P. austriacus* has a narrower trophic niche than *P. auritus*. This higher degree of dietary specialization may force *P. austriacus* to cover greater distances to appropriate foraging habitats compared to *P. auritus*. The latter species seems to be more generalistic in its dietary patterns and might be able to find a wider range of potential foraging sites in the vicinity of its roosts. This suggests that *P. austriacus*, as a more specialized species, has to spend more time for food acquisition in comparison to *P. auritus*. In addition, the seasonal presence of the two species in the study area may also play an important part in the resource partitioning [77]: roost site monitoring indicates that the activity of *Plecotus austriacus* (May to the beginning October) is delayed relative to *P. auritus* (April to the end of October) in spring and declined earlier in autumn. However, these phenological differences are probably also related to the species’ respective diets. 

Our reconstruction of the species’ diets lead to the assumption that both species generally feed on the same prey categories and therefore have a similar niche breadth. This applies with regard to numerical proportion and volume proportion of detected prey items. However, we could identify differences with regard to frequency of prey items: in nearly every sample of *P. austriacus* we found evidence for consumed Lepidoptera, while in the samples of *P. auritus,* only in 70% of the samples. This indicates that *P. austriacus* exhibits a lower dietary niche breadth in comparison to *P. auritus*. Pianka’s overlap index of 0.86 suggests that the diets of *P. auritus* and *P. austriacus* may be less similar than previously thought. Moreover, seasonal niche breadth is more variable among samples of *P. auritus* compared to samples of *P. austriacus*. Significant seasonal changes in the diet composition of insectivorous bats may indicate flexible exploitation of available food resources, e.g., [47,77,78], certain foraging opportunism, and less selective feeding [79,80,81,82,83]. At the same time, the lower niche breadth of *P. austriacus* indicates a high vulnerability to a potential loss of foraging habitats. In our study, the analysis of fecal samples from roosting sites that allowed exact assignment with the two species revealed subtle differences in the diet; the presence of non-volant arthropods within the diet of *P. auritus*, such as Arachnida, Chilopoda, and Dermaptera, confirms the use of gleaning as a feeding style [27,84]. In contrast, fewer prey categories were detected in the fecal samples of *P. austriacus*, since all of these prey items were volant arthropods. 

Further, we were able to detect seasonal differences with regard to a prey category used by both species; the lepidopteran families Saturniidae and Lymantriidae make up a notable proportion in the diet of *P*. *auritus* during the reproductive season. In contrast, moths of the family Cymatophoridae seem to make up a notable proportion in the diet of *P. austriacus* during spring. Nocturnal moths of the family Arctidae are preyed upon by both species mainly in autumn. However, actual diet composition is also heavily influenced by actual food supply and availability, and its seasonal fluctuations. Thus, our results are only “a snapshot in time”. There could well be a greater but also a lesser similarity between the two species. In addition, morphological fecal analysis can determine prey organisms only to the order or family level which limits the significance of dietary studies using this method. However, while rare or highly digestible prey is often underrepresented using microscopic identification, volume and proportion of the diet can be examined accurately [85]. Moreover, diets of bats may vary within species across different regions [47]. Therefore, measuring the diets of bat species from feces collected from different regions within the distribution range, a high sample number across different seasons, and supplemented by advanced analysis techniques (e.g., high throughput sequencing [5] and DNA-metabarcoding [86]) will be directly of use for future conservation strategies.

Finally, an insectivorous bat’s spatial behavior and habitat use depends largely on its sensory ability to retrieve food. Its ability to capture a prey item is determined by its flight capabilities, particularly agility and maneuverability [32], which in turn are influenced by wing morphology [66,87] and body size [87]. Changes in morphology result in differences in flight performance [88,89], which directly affect habitat use [89,90,91]. Differences in wing morphological features, such as wing loading, can be significant predictors of habitat use [92]. However, similarity in wing morphology does not necessarily restrict species to similar foraging behaviors or similar habitats [93]. In closely-related and phenotypically-similar species, there may be fine modifications of a given suite of features or wing construction rather than dramatic differences [90,93,94,95]. Our data support the available information in the literature, indicating that the two species are highly similar in several external features, including body size and body mass. Accordingly, we identified only marginal phenotypic differences between the two species. These minor differences are not sufficient to explain the striking differences in behavior in a plausible way. Rather, the flexibility in habitat use observed in this study might underlie the ability of individuals to exploit different resources in different environments, so that the same species should be able to realize different niches when being confronted with spatially distinct resource distributions. In addition, interspecific competition may be an important driver of the two species’ distribution at small spatial scales: within our study, *P. auritus* and *P. austriacus* occur in the same habitat but may have evolved different preferences towards distinct subsets of the limiting resource, resulting in considerable changes in habitat use accompanied with divergence in their habitat preferences in sympatry. Resource partitioning seems therefore to be the main driver of their ecological differentiation, which ultimately allows these cooccurring species to coexist in their environment.

Our results imply differences in the ecological requirements of *P. auritus* and *P. austriacus*. These differences give reason to reflect the conservation needs for these sympatric long-eared bat species (Table 2). This seems even more important, as *Plecotus austriacus* is reported to have a presumably negative population growth [96,97,98,99], and thus is likely to become a severely endangered species as is already the case in other parts of Europe [100]. Although the preservation of buildings as possible roosting structures is immanently important, this study shows that not only the protection of roosts may promote the conservation of local populations, but also that the protection of existing or the creation of new hunting habitats is an important strategy for ensuring the functionality of the overall habitat of a species. *P. auritus* is dependent on forests for both roosting and foraging and priority should be given to woodland management for this species (Table 2). For *P. austriacus*, current efforts for the conservation of colonies must go far beyond the preservation of actual roosts and in particular take into account the numerous foraging habitats in agricultural areas and in settlements (Table 2). Recently, it has been demonstrated that structural complexity of habitats with features relevant to bats for commuting, foraging, and roosting may be influenced by specific management practices within different types of land use [28]. For this reason, future conservation efforts for these two species should be oriented towards an improved adaptation in forestry and agricultural measures.

Summarizing, we strongly emphasize the need for improved, more species-specific conservation measures for *P. auritus* and *P. austriacus* based on a better understanding of associated factors (e.g., land use) that threaten the respective species. We conclude that contact zones of sympatrically occurring bat species are especially interesting as they can enhance our understanding of the coexistence of similar species. In the case of *P. auritus* and *P. austriacus,* the question arises regarding whether there might be differences between allopatric and sympatric populations. Given this, we generally stress the urgency for a greater number of studies looking into the temporal, seasonal, and life history variations between closely-related bat species in syntopy. 

## Figures and Tables

**Table 1:** Spatio-temporal behavior of female radio-tracked *Plecotus auritus* (*n* = 5) and *Plecotus austriacus* (*n* = 5). Values for each parameter are presented as mean ± SD; 100% MCP areas are given in area total (over full tracking period) and per night; core areas = total area of all 85% cluster cores over the radio-tracking period; * significant differences (*p* ≤ 0.005).

	*P. auritus*	*P. austriacus*
Activity area total (100% MCP)/ha *	544.54 ± 295.89	2828.3 ± 1269.43
Activity area per night (100% MCP)/ha *	101.46 ± 71.33	548.19 ± 85.57
Core area (85% clusters)/ha *	49.2 ± 25.6	149.7 ± 0.07
Travel distance per night/km *	4.23 ± 2.8	12.72 ± 3.7
Max.nightly distance to foraging areas/km *	2.01 ± 0.78	6.16 ± 2.12
Number of foraging areas	7.2 ± 1.3	9.54 ± 1.52
Number of roosts	2.4 ± 1.14	1
Emergence/min after sunset	30 ± 10	25 ± 15
Foraging time/min *	311 ± 135	347 ± 117
Night roosting/min *	40 ± 20	25 ± 10

**Table 2:** Identified priority conservation needs for *Plecotus auritus* and *Plecotus austriacus*.

*Plecotus auritus*	*Plecotus austriacus*
Preservation of standing dead trees and trees with cavities or crevice roosts in older stands	Maintance or creation of hedgerows, rows of trees, copses and hedge strips as connecting lines of widely dispersed foraging habitats
Establishment of network of biotope tree candidates in younger stands (future trees/Z-trees)	Preservation of remaining unimproved grasslands and foster availability of new foraging habitats
Establishment of smaller, but well-connected, forest areas with deciduous and mixed stands near water bodies as hunting habitats (ideally primeval beech forests, with a mosaic of optimal stages (beech forest), decaying stages (patchy crown layer) and growing stages (multi-layered structure))	Increase the availability of unmanaged field margins at arable or pasture field edges through management practices and promotion of structurally rich village edges (e.g., meadow orchards)
Optimization of forest edges (graded woodland fringes, flower-rich inner forest edges, shrub-rich outer forest edges) and forest meadows, loose multi-layered forests gaps and light shafts	Inclusion of and cooperation with land owners, land managers, farmers, and roost owners to increase awareness and identify roosts in buildings
Preservation of old buildings, especially in small dispersed settlements	
Avoidance of fertilizers and agrochemicals on grassland sites by farmers but also in private gardens in rural settlements	
Leaving standing and lying dead wood in any relevant bat habitat to increases insect diversity	
Reconstruction of wetland sites or creation of new wetland biotopes to establish productive foraging habitats.	
Consideration of differential arrival at and departure from maternity colonies with regard to renovation projects of buildings (e.g., renovation of churches) in the scheduling of construction measures (start and end of renovation measures).	

## Data Availability

No new data were created or analyzed in this study. Data sharing is not applicable to this article.

## Supplementary Materials

The following are available online at https://www.mdpi.com/article/10.3390/ani11123460/s1, Table S1: Mean ± SD (min–max) values for measurements of morphological characters used for field identification and calculated wing morphometry of captured individuals of *P. auritus* (*n* = 51) and *P. austriacus* (*n* = 26); * significant (*p* < 0.005) and ** highly significant (*p* < 0.001) variation between species.
