# Supplementary material for: Spatial Behavior and Habitat Use of Two Sympatric Bat Species
Source: Animals (Basel). 2021 Dec 5;11(12):3460. doi: 10.3390/ani11123460 (PMC8697949; doi:10.3390/ani11123460)
Supplement: Supplementary file 1 [file animals-11-03460-s001.zip › animals-1469441-supplementary.pdf]

Supplement

Table S1. Mean ± SD (Min−Max) values for measurements of morphological characters used for field identification and calculated wing morphometry of captured individuals of *P. auritus* (n = 51) and *P. austriacus* (n = 26); * significant ($p \leq 0.005$) and ** highly significant ($p \leq 0.001$) variation between species.

	Plecotus auritus		***Plecotus austriacus***	
	mean ± SD	***min−max***	***mean ± SD***	***min−max***
Head-Body-Length, *HBL* [mm]	52.43 ± 4.95	42.0−59.0	54.34 ± 3.84	42.5−60.5
Thumb length, *TL* [mm]*	6.79 ± 0.45	5.5−7.1	6.16 ± 0.58	5.4−7.1
Claw length, CL [mm]*	2.55 ± 0.28	1.9−3.0	1.84 ± 0.33	1.5−2.1
Hind foot length, *HF* [mm]	8.3 ± 1.19	6.9−10.6	8.24 ± 0.59	7.0−9.0
Ear length, *EL* [mm]	36.77 ± 2.88	26.2−38.5	36.92 ± 2.91	28.6−41.0
Tragus length, *TrL* [mm]*	15.57 ± 0.42	14.9−16.2	15.86 ± 2.97	14.5−17.0
Tragus width, *TrW* [mm]*	4.91 ± 0.38	4.2−5.5	5.93 ± 0.18	5.5−6.3
Body mass, *m* [g]	8.97 ± 1.03	6.9−10.8	9.49 ± 0.04	7.9−11.0
Forearm length, *FA* [mm]	40.66 ± 2.1	35.1−43.2	42.1 ± 1.64	37.0−43.2
Length of 3rd finger, *D3* [mm]**	65.48 ± 0.98	64.0−67.0	68.48 ± 2.23	64.5−71
Length of 5th finger, *D5* [mm]**	53.6 ± 1.3	49.0−55.0	52.68 ± 1.26	49.0−54.5
Wing span, *WS* [mm]*	259.61 ± 7.1	240.0−271.0	268.34 ± 5.95	255.0−281.0
Wing area, *S* [m^2]*	0.0093 ± 0.0003	0.0084−0.0099	0.0093 ± 0.0004	0.0083−0.01
Aspect ratio, *AR***	7.29 ± 0.37	5.97−7.85	7.73 ± 0.29	6.9−8.14
Wing loading, *WL* [N/m^2]**	8.61 ± 0.78	6.84−10.9	9.98 ± 0.84	8.37−11.61
